# Dissociation of Clinical Outcomes and CSF Proteinopathy Biomarkers in Parkinson’s Disease: Cognitive–Affective Dissociation with Specificity for Tau

**DOI:** 10.3390/biomedicines13102478

**Published:** 2025-10-11

**Authors:** João Paulo Mota Telles, Lucas Camargo, Anna Carolyna Gianlorenço, Felipe Fregni

**Affiliations:** 1 Department of Neurology, University of São Paulo, São Paulo 01246-903, SP, Brazil; 2 Spaulding Neuromodulation Center and Center for Clinical Research Learning, Spaulding Rehabilitation Hospital, Harvard Medical School, Boston, MA 02138, USA; 3 Laboratory of Neuroscience and Neurological Rehabilitation, Physical Therapy Department, Federal University of São Carlos, São Carlos 13565-905, SP, Brazil

**Keywords:** Parkinson’s disease, alpha-synuclein, amyloid-beta, tau proteins, cognition, mood

## Abstract

**Background:** Parkinson’s disease (PD) is characterized not only by motor dysfunction but also by widespread degeneration across cortico-striatal, limbic, and cortical circuits. Mounting evidence suggests that tau and α-synuclein pathology underlie these processes, though how these proteinopathies translate into affective and cognitive outcomes remains uncertain. Depression and anxiety are highly prevalent in PD, yet the biological correlates of these affective disturbances are poorly defined. **Methods:** This is a retrospective analysis of existing data from the Parkinson’s Progression Markers Initiative (PPMI). Montreal Cognitive Assessment (MoCA), geriatric depression scale (GDS), and State-Trait Anxiety Inventory (STAI) were used to assess cognition, depression, and anxiety in PD, respectively. The CSF biomarkers evaluated were Aβ42, t-tau, and p-tau181, using Elecsys electro-chemiluminescence immunoassays on the cobas e601 platform (Roche Diagnostics). **Results:** From the 4380 patients who had GDS information, the MoCA test was collected from 438 patients, and 445 from the GDS test for depression, and the STAI screening for anxiety. There were no significant differences in biomarker levels between patients with depression (GDS ≥ 5) and those without (GDS < 5), nor between patients with anxiety (STAI > 40) and those with lower anxiety scores (STAI ≤ 40). In contrast, cognitive outcomes showed clear associations. Patients with cognitive impairment (MoCA < 26) demonstrated higher levels of pTau (*p* = 0.02) and tTau (*p* = 0.01), as well as elevated pTau/Aβ42 (*p* = 0.003) and tTau/Aβ42 (*p* = 0.002) ratios compared to those with MoCA ≥ 26. In multivariate analysis, both pTau/Aβ42 > 0.022 (OR 4.64, 95% CI 1.67–13.8) and tTau/Aβ42 > 0.26 (OR 4.18, 95% CI 1.6–11.5) remained significantly associated with cognitive decline. In a longitudinal analysis in the first 3 years of follow-up, cognition in PD remained lower than in controls, while CSF p-tau and Aβ42 remained higher in controls. **Conclusions:** In our cohort, no associations were found between CSF biomarkers and depression or anxiety, underscoring that mood disturbances in PD are likely mediated by alternative mechanisms such as monoaminergic dysregulation, neuroinflammation, and psychosocial factors. By contrast, cognitive performance (MoCA) was clearly linked to tau-related pathology, rather than α-synuclein or Aβ42 alone. While Aβ42 and α-synuclein remain useful for staging and assessing global disease risk, our findings highlight the specificity of tau-related pathology for cognitive outcomes in PD.

## 1. Introduction

Parkinson’s disease (PD) is a progressive neurodegenerative disorder primarily characterized by motor symptoms such as tremor, rigidity, and bradykinesia [1]. However, a broad range of non-motor symptoms are also present and can even precede clinical diagnosis by years [2]. Among these, depression and anxiety are the most prevalent psychiatric comorbidities, while cognitive impairment is common and frequently progresses to dementia [2,3]. Notably, approximately 80% of individuals with PD develop dementia in the long term, after 15 to 20 years [4], while up to 50% present depressive symptoms [5], substantially increasing the morbidity and reducing their quality of life [6,7]. Identifying biological correlates of cognitive and mood disorders in early-stage PD is challenging due to the subjective nature of these symptoms, which can fluctuate over days or years in response to intrinsic factors and external influences such as family support, social engagement, and environmental conditions [6,8,9]. Thus, establishing biomarkers that reflect the neuropathological processes is critical for early detection, risk stratification, and the development of targeted interventions to mitigate long-term neuropsychiatric burden for patients and caregivers.

Several molecular markers have been implicated in PD progression and non-motor symptomatology [10,11]. Among them, the Apolipoprotein E (ApoE) genotype, particularly ε4, is known to influence amyloid and tau accumulation, contributing to cognitive and emotional dysfunction [12]. Alpha-synuclein (α-synuclein) aggregates constitute PD’s pathological hallmark, as previous studies indicate it has a role in modulating the dopamine (DA) release in the striatal region, by increasing intracellular Ca^2+^, disrupting neuronal intracellular homeostasis, resulting in a neurotoxicity cascade and neurodegeneration [13,14,15]. Clinically, this neurodegenerative process in the limbic networks and prefrontal areas results in cognitive decline and mood changes [16,17,18].

Furthermore, Tau—both total (t-tau) and phosphorylated (p-tau) forms—and β-amyloid (Aβ42) abnormalities, classically associated with Alzheimer’s disease (AD), are increasingly recognized in PD, particularly in those with non-motor symptoms [11]. Tau—as a neuronal microtubule-associated protein—is responsible for anterograde and retrograde axonal organelles transport, and for stabilizing β-catenin, thus protecting the neuron from acute apoptosis [19,20,21]. In patients with AD, tau phosphorylation is increased in comparison with healthy controls [22], which could be one of the mechanisms of the neurodegenerative process and reduced cognitive performance in those patients [23]. In the context of PD, the increase in tau and tau/Aβ42 in CSF indicates a correlation with cognitive impairment in PD subjects, but the results in the literature are still mixed [24,25,26].

Depression and anxiety are among the most prevalent non-motor symptoms in Parkinson’s disease, affecting nearly half of patients over the disease course [2,3]. Importantly, several studies suggest that depressive symptoms may not only co-occur with motor dysfunction but also precede both motor and cognitive manifestations [1], in some cases emerging years before a formal PD diagnosis. This early onset highlights the potential role of affective disturbances as prodromal markers of neurodegeneration or a common mechanism leading to degeneration, such as neuroinflammation [12]. Despite their frequency and early presentation, the biological underpinnings of these affective disorders in PD remain poorly understood, underscoring the need to clarify whether they share mechanistic links with protein aggregation processes or reflect distinct neurochemical and psychosocial pathways.

While each marker contributes to PD pathophysiology, their combined relationship to depression, anxiety, and cognitive impairment in PD remains poorly understood. Therefore, this study aimed to evaluate the association of depression, anxiety, and cognitive impairment in patients with PD with cerebrospinal fluid (CSF) biomarkers, particularly α-synuclein, Aβ42, total Tau, and phosphorylated Tau. By identifying biomarkers associated with neuropsychiatric impairment could improve early detection and the development of personalized targeted treatments.

## 2. Methods

This is a cohort retrospective analysis of existing Tier 1 data from the Parkinson’s Progression Markers Initiative (PPMI) [27], a centralized an open-access repository from longitudinal studies to identify and validate biomarkers for PD onset and progression. Data used in the preparation of this article was obtained on 2 August 2025 from the Parkinson’s Progression Markers Initiative (PPMI) database (www.ppmi-info.org/access-dataspecimens/download-data), RRID:SCR_006431. For up-to-date information on the study, visit www.ppmi-info.org.

From this data, we included sporadic PD participants (drug-naïve or early treated), with available CSF data, cognitive and mood assessments. Exclusion criteria were (i) monogenic PD (LRRK2, GBA, SNCA carriers); (ii) missing clinical assessments or biomarker data. Collected data included age, sex, ethnicity, and education years.

### 2.1. Outcomes

The primary goal of this study was to analyze cognitive performance and mood changes in PD patients and investigate associations between those clinical features and CSF biomarkers. Cognition was assessed by the Montreal Cognitive Assessment (MoCA), with values < 26 indicating cognitive impairment and ≥26 indicating normal cognitive performance [28]. Depression was evaluated using the 15-item geriatric depression scale (GDS) [29], with values ≥ 5 indicating depression, and ≥9 indicating moderate-to-severe depression. A secondary goal was to analyze anxiety, which was evaluated using the State-Trait Anxiety Inventory (STAI) [30].

### 2.2. Biomarkers

The CSF biomarkers evaluated were A-42, total tau (t-tau), and phosphorylated tau at the threonine 181 position (p-tau181), using Elecsys electro-chemiluminescence immunoassays on the cobas e601 platform (Roche Diagnostics) [31,32]. Cutoffs for tau/Aβ ratios were obtained from previous literature [33].

In order to validate biomarkers, their levels were compared between patients with PD and healthy controls from the same PPMI cohort. Then, biomarkers were compared between patients with and without cognitive decline, defined based on the Montreal Cognitive Assessment (MoCA) test score, since cognitive decline has a well-established association with amyloid biomarkers, both for the general population and PD patients [28,34].

### 2.3. Statistical Analysis

Continuous variables are described as mean (±standard deviation) or median (interquartile range), and use visualization tools (histograms, box plots, and scatter plots) to visually inspect the data for normality checks. Categorical variables are described as frequency (valid %). Hypothesis tests were performed with *t*-tests, Wilcoxon tests, Fisher’s exact test, or chi-squared tests, as appropriate. Correlations were tested using Pearson’s product-moment correlation coefficient. Whenever there were multiple assessments of the biomarkers or the GDS, the first one was extracted for analysis. Values below the test detection threshold were approximated to 0 for analysis purposes. Biomarkers are compared between groups using *t*-tests and logistic regressions, both continuously and as dichotomous variables. Logistic regressions were modeled for the primary (cognition) and secondary (depression and anxiety) outcomes only if hypothesis tests were significant, to avoid unnecessary multiple testing. Variables showing *p* < 0.20 in univariable analyses were included together in a multivariable logistic model to identify independent predictors of cognition, depression, and anxiety. Results were reported as odds ratios (OR) with 95% confidence intervals and *p*-value.

Longitudinal analysis was performed considering 4 time-points: Baseline (BL), V4 (year 1), V6 (year 2), V8 (year 3). Data that was not collected at BL, but at screening, was analyzed with BL. We performed linear mixed-effects models (LMMs) with a random intercept for participant: outcomes were the MoCA, GDS, STAI, and CSF biomarkers (p-tau, t-tau, Aβ1-42, and the p-tau/Aβ1-42 ratio), considering within-person correlation from repeated measurements. Time was modeled as a categorical factor using the scheduled visits (BL, V04–V08), Group as PD vs. Control, and primary fixed effects were Group, Time, and their interaction (Group × Time For biomarker evolution, biomarkers was natural-log transformed and we used a within–between decomposition of log-biomarker values: models included log-biomarker × Time, with Group × Time and a participant random intercept, and log ratio, computed as log p-tau − log Aβ1-42. Planned contrasts compared follow-up visits to Baseline.

Statistical significance was set at two-tailed *p* < 0.05. A repository with relevant code is available at https://zenodo.org/records/17012543 (Accessed on 28 August 2025). All analyses were conducted using R (R Foundation for Statistical Computing, Version 2022.07.1, Vienna, Austria, 2024).

## 3. Results

### 3.1. Patient Characteristics

From the 4380 patients who had GDS information, 438 patients completed the MoCA test, and population characteristics according to cognitive status are shown in Table 1. Patients with cognitive impairment (MoCA < 26) were older (64.3 ± 9.0 versus 59.1 ± 9.0 years, *p* < 0.001), had fewer years of education (14.8 ± 4.0 versus 16.2 ± 3.3, *p* < 0.001), a higher proportion of non-White ethnicity (7.5% versus 1.0%, *p* < 0.001), and slightly higher Geriatric Depression Scale scores (5.9 ± 1.7 versus 5.4 ± 1.6, *p* = 0.008). Sex distribution, motor severity (MDS-UPDRS III), anxiety levels (STAI), and LRRK2 mutation status did not differ between groups.

A total of 445 subjects who completed the GDS test for depression are shown in Table 2. Patients with positive depression screening (GDS ≥ 5) had fewer education years (15.6 ± 3.7 versus 16.7 ± 3.1, *p* = 0.003), higher STAI scores (47.5 ± 5.2 versus 45.1 ± 5.9, *p* < 0.001), and lower MoCA scores (26.2 ± 3.4 versus 26.9 ± 2.2, *p* = 0.01). Age, sex, and ethnicity did not differ between groups.

445 subjects who completed the STAI screening for anxiety are presented in Table 3. Patients with positive anxiety screening (STAI > 40) displayed slightly better cognitive performance (MoCA 26.5 ± 3.1 versus 25.1 ± 3.8, *p* = 0.02), while age, sex, ethnicity, education, motor impairment (MDS-UPDRS III), and LRRK2 status were similar between anxiety-positive and anxiety-negative groups.

### 3.2. Biomarker Description and Validation

Mean CSF biomarker values were 100.62 (± 47.0) pg/mL for α-synuclein, 14.09 pg/mL for pTau, 163.0 (± 55.5) pg/mL for tTau, and 942.2 (± 397.8) pg/mL for Aβ-42. A total of 217 (72.3%) of people had Aβ-42 levels below 1100 pg/mL, 19 (6.9%) had pTau/Aβ-42 ratio > 0.022, and 21 (7.2%) had tTau/Aβ-42 ratio > 0.26.

Biomarkers were significantly different between patients with PD and healthy controls, except for Aβ-42 (Table 4), including lower α-synuclein levels and higher frequencies of pTau/Aβ-42 and tTau/Aβ-42 ratios. There was no correlation between α-synuclein levels and MDS UPDRS III total scores (Pearson Correlation, *p* = 0.95).

### 3.3. Cognition, Depression and Anxiety

**Figure 1:**
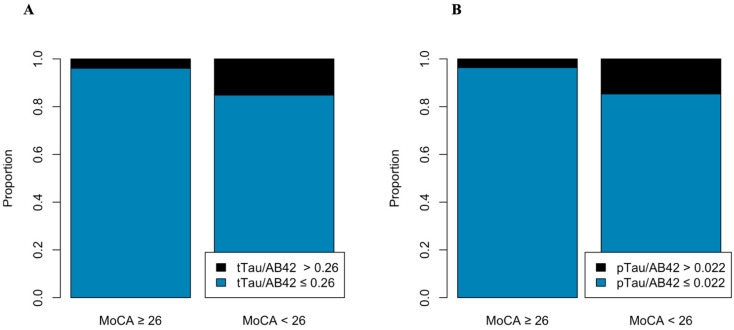
pTau/Aβ-42 and tTau/Aβ-42 differ between groups according to cognitive decline (MoCA < 26). (**A**) pTau/Aβ-42 > 0.022 and (**B**) tTau/Aβ-42 > 0.26 are suggestive of amyloid pathology, with amyloid deposition and tau increase. Patients with cognitive decline (MoCA < 26) had significantly higher rates of both ratios (*p* = 0.003 and *p* = 0.002).

Cognitive performance assessed by MoCA demonstrated statistically significant differences in pTau (*p* = 0.02), tTau (*p* = 0.01), and pTau/Aβ-42 (*p* = 0.003) and tTau/Aβ-42 (0.002) ratios between MoCA ≥ 26 and MoCA < 26 groups, see Table 5. Additionally, pTau/Aβ-42 > 0.022 (OR 4.64, 95%CI 1.67–13.8) and tTau/Aβ-42 > 0.26 (OR 4.18, 95%CI 1.6–11.5) were significantly associated with cognitive decline (MoCA < 26), even after adjustment for age and education years, see Table 6, Figure 1.

**Figure 2:**
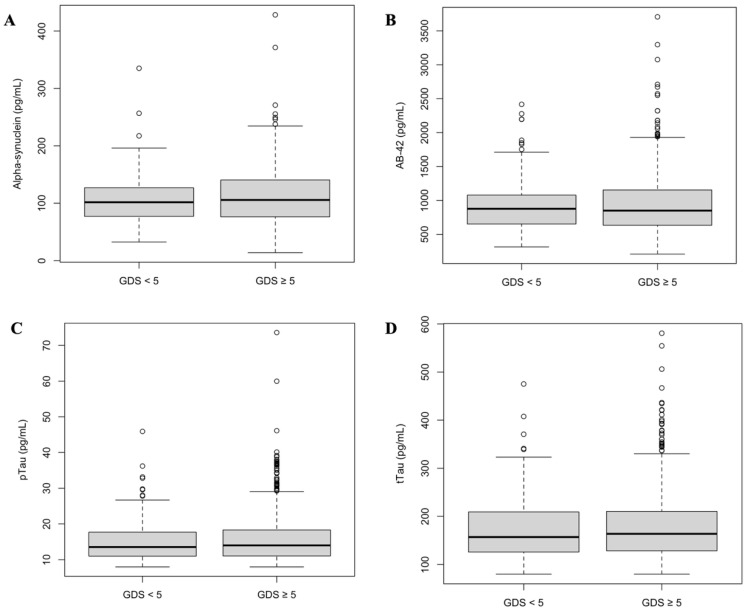
Biomarker levels and GDS scores. There were no difference in biomarker levels comparing patients with positive depression screening (GDS ≥ 5) and negative screening (GDS < 5). There were no significant differences between groups in (**A**) alpha-synuclein (*p* = 0.27); (**B**) Aβ-42 (*p* = 0.62); (**C**) pTau (*p* = 0.25); and (**D**) tTau (*p* = 0.57).

Depression scores demonstrated no correlation between the continuous values of α-synuclein and GDS scores (*p* = 0.53), Aβ-42 (*p* = 0.29), pTau (*p* = 0.60), or tTau (*p* = 0.66), as well as in biomarkers between patients with GDS ≥ 5 and GDS < 5 (Table 7, Figure 2), both when analyzing continuous biomarker levels and dichotomized levels and ratios. There were also no differences when comparing groups with GDS ≥ 9 or GDS < 9.

Furthermore, in our multivariate analysis, no statistically significant associations were observed between (GDS ≥ 5) and α-synuclein (OR 1.005, 95%CI 0.997–1.014), pTau (OR 0.99, 95%CI 0.94–1.04), tTau (OR 0.997, 95%CI 0.993–1.002), Aβ-42 (OR 0.999, 95%CI 0.999–1.001), α-synuclein > p75 (OR 2.03, 0.75–6.54), Aβ-42 < 1100 (OR 0.98, 95%CI 0.53–1.78), pTau/Aβ-42 > 0.022 (OR 2.03, 95%CI 0.64–9.08), or tTau/Aβ-42 > 0.26 (OR 1.48, 95%CI 0.51–5.36), see Table 8. Similarly, no associations between a positive anxiety screening and α-synuclein (*p* = 0.97), pTau (*p* = 0.95), tTau (*p* = 0.87), Aβ-42 (*p* = 0.9), α-synuclein > p75 (*p* 0.69), Aβ-42 < 1100 (*p* = 1.0), pTau/Aβ-42 > 0.022 (*p* = 0.57), or tTau/Aβ-42 > 0.26 (*p* = 0.48) was detected, see Table 9.

### 3.4. Longitudinal Cognition and CSF Biomarkers

Using linear mixed-effects models with random intercepts for participants and fixed effects for group (PD vs. Control), visit, and their interaction, we analyzed repeated MoCA scores and CSF biomarkers from BL through V8 (MoCA: PD *n* = 703, controls *n* = 207; biomarkers: p-tau PD *n* = 487/controls *n* = 176; t-tau PD *n* = 487/controls *n* = 176; p-tau/Aβ42 PD *n* = 486/controls *n* = 176). We observed a robust group versus time interaction for MoCA (*p* < 0.001, Figure S1). Although the baseline PD–control difference was imprecise and not statistically significant (difference = +2.35 points favoring controls; SE = 3.07; *p* = 0.44), controls performed consistently better at follow-up, with between-group differences of approximately 1.14 points at V04 (*p* < 0.001), 1.33 points at V06 (*p* < 0.001) and 1.25 points at V08 (*p* < 0.001). Marginal means suggested small declines from BL to follow-ups in both groups (controls 29.0 to 27.5; PD 26.7 to 26.2), yielding a persistent post-baseline gap of 1.1–1.3 MoCA points.

CSF biomarkers demonstrated clear temporal change and group differences (Figure S2). On the log scale, controls had higher p-tau than PD at every visit (main effect of group *p* < 0.001; time *p* < 0.001; group × time *p* < 0.001). The control–PD contrasts ranged from 0.14 to 0.24 log-units from BL to V08, corresponding to approximately 15–28% higher p-tau in controls (e.g., BL: +15%, V08: +24%). Aβ42 concentrations were likewise higher in controls (group *p* < 0.001; time *p* < 0.001; group × time *p* < 0.001), with differences of 0.12–0.19 log-units (+12–21%). For the p-tau/Aβ42 ratio, we also detected significant group × time interaction (group *p* = 0.084; time *p* < 0.001; group × time *p* < 0.001): PD exhibited a slightly lower ratio at BL (−2.2% vs. Controls), convergence by mid-follow-up, and a modest elevation by V08 (+1.8%).

## 4. Discussion

This retrospective study analyzed data from over 1000 patients with PD who had measurements of α-synuclein, Aβ42, and tau-related CSF biomarkers and their association with cognition, depression, and anxiety symptoms. We observed that PD subjects with cognitive decline (MoCA score below 26) demonstrated higher levels of pTau, tTau in comparison with those with a MoCA score above 26. The same patterns were observed when comparing the pTau/Aβ-42 and tTau/Aβ-42 ratio increased in those patients with lower cognitive performance. These results indicate that the accumulation of those biomarkers in the CSF indicates a more advanced neurodegenerative process and dementia. However, those biomarkers could not detect changes associated with depression and anxiety scores, suggesting different neurophysiological mechanisms of those psychiatric conditions in this population. At follow-up, cognition in PD remained lower than in controls by 1.1–1.3 MoCA points—statistically robust but modest in magnitude—while biomarker profiles differed consistently between groups and evolved over time (higher p-tau and Aβ42 in controls; a narrowing and slight reversal in p-tau/Aβ42 by V08). Baseline MoCA contrasts were accompanied by large standard errors and should be interpreted with caution.

### 4.1. Cognitive Impairment in PD

The characteristics of cognitive impairment in PD subjects vary in severity and progression rate, in which memory loss is not necessarily present in the early stage, but other cognitive domains like visuospatial and executive functions are affected more prominently than the normal aging processes [4,35]. The physiopathology of PD and the cognitive decline in this population is strongly related to the presence of Lewy bodies (LB), an aggregation of intracellular vesicles and protein α-synuclein, as a result of the inappropriate intracellular traffic of organelles and proteins pathway [36,37]. Many questions remain regarding how the intracellular toxicity of α-synuclein leads to neuron loss and neurodegeneration; however, evidence has proved that its aggregation is a primary component of LB [38].

Some researchers argue that the aggregation of α-synuclein per se does not seem to represent neurotoxicity, but it is rather a protective mechanism against an impaired degradation of α-synuclein through activation of the autophagy-lysosomal pathway (ALP) [11,38,39]. Models suggest that the formation of LB is a consequence of dysfunctional proteasome activity and impaired endoplasmic reticulum vesicle trafficking with the Golgi network [40,41]. LBs are implicated in dopaminergic neuronal loss, which underlies the cardinal motor manifestations of PD [26]. The Lewy Body Disease (LBD) spectrum includes dementia with Lewy bodies (DLB) and Parkinson’s disease dementia (PDD); these entities share overlapping pathology and clinical features but are distinguished clinically by timing: cognitive impairment that precedes or occurs within approximately 1 year of parkinsonism suggests DLB, whereas dementia that develops after well-established PD supports PDD [42,43].

Our findings are consistent with the literature, demonstrating reduced CSF α-synuclein in PD patients in comparison to healthy controls [44,45], but we could not detect differences between PD subjects with normal and reduced cognitive scores. On the other hand, p-tau, t-tau, pTau/Aβ-42 and tTau/Aβ-42 ratios are increased in PD individuals with cognitive impairment. Despite the fact that, in our multivariate models the correlation loses statistical significance for Tau proteins after adjusting by age and education years, the correlation with pTau/Aβ-42 and tTau/Aβ-42 ratios remained statistically significant. Interestingly, tau protein and Aβ42 are known to interact with α-synuclein, promoting the neurodegenerative process and accelerating cognitive dysfunction [46]. Palmio et al. (2009) [47] suggest that increased tau proteins in the CSF could indicate neuronal damage. Additionally, Liu et al. (2015) [25] suggest that higher p-tau and pTau/Aβ-42 are associated with reduced cognitive performance in PD. Postmortem studies demonstrated the presence of Tau and α-synuclein in LB, indicating a synergic activity in the neurodegenerative process and cognitive decline [48], therefore signaling that α-synuclein in LB may be the driver of this process, not in the CSF.

Moreover, other studies observed that the presence of Tau and Aβ plaques is more associated with cognitive decline in PD than α-synuclein [49,50]. Experimental models further indicate a bidirectional cross-seeding loop in which Aβ fibrils accelerate tau aggregation and hyperphosphorylation, while tau oligomers subsequently facilitate secondary α-synuclein misfolding, amplifying toxicity across connected cortical circuits [51,52]. Collectively, tau and Aβ act as synergistic drivers of neurodegeneration in PD, providing a mechanistic substrate for the cognitive impairment seen in patients with mixed protein-aggregation disorder [11,53,54].

### 4.2. Depression and Anxiety in PD

Depression and anxiety symptoms are present in approximately 50% patients with PD [55], and major depressive disorder is observed in up to 20% of these patients, with a significant impact on their quality of life [56]. The neurobiology and pathogenesis of depression and anxiety are multifactorial and complex [24,57]. The low levels of serotonin (5-HT), alone, are not enough to cause depression symptoms, but a combined alteration in other monoamines, such as norepinephrine (NE) and DA, is also necessary [24]. Chronic stress directly impacts the dysregulation of NE release by stimulating the secretion of corticotropin-releasing factor in the hypothalamus, upregulating the pituitary gland to increase levels of ACTH, resulting in higher levels of NE and cortisol release by the adrenal glands [58]. Altered cortisol levels cause inappropriate cytokine activity and inflammatory response with neurotoxicity effects, alongside many other mechanisms (such as neural connectivity alterations, anatomic abnormalities, and cellular dysfunction) [59].

Additionally, chronic stress factors also cause disturbances in the dopaminergic mesolimbic pathway in the ventral tegmental area, with projections to the nucleus accumbens associated with the reward pathway and mood [60]. Moreover, the overexpression of α-synuclein in dopaminergic neurons results in neuronal death [61]. This impairment in the dopaminergic and reward systems is known to be associated with psychiatric symptoms in neurodegenerative disorders such as PD. Therefore, there is intense research seeking to identify adequate biomarkers for depression and anxiety, as this could lead to a greater insight into symptoms onset, relapse, and recurrence, even possibly enabling the development of pathogenesis-driven treatments [62]. Clearly, mental health issues in PD are not restricted to only neurotransmitter synaptic imbalance, but also socioeconomic factors—like concerns about the future, family and friends support—which intensify with the progression of the disease, along with physical and cognitive limitations, increasing the vulnerability of this population to psychiatric disorders [8].

The dissociation between affective and cognitive outcomes in relation to CSF proteinopathy biomarkers underscores the complexity of underlying neural mechanisms and suggests that distinct pathological processes may drive changes across separate brain circuits. For instance, dopaminergic dysfunction and α-synuclein accumulation may preferentially disrupt frontostriatal pathways linked to mood regulation, whereas tau or amyloid pathology may exert greater effects on cortico-hippocampal networks involved in cognition [11]. Supporting this view, a study comparing sertraline with repetitive transcranial magnetic stimulation (rTMS) of the left dorsolateral prefrontal cortex (DLPFC) demonstrated that while both interventions alleviated depressive symptoms, only rTMS led to measurable improvements in cognitive performance [63]. This dissociation reinforces the notion that pharmacological and neuromodulatory interventions may target overlapping yet distinct neurobiological substrates. Taken together, these findings highlight the importance of tailoring treatment strategies in Parkinson’s disease (PD), not only by symptom domain but also by the underlying circuitry affected, and they underscore the potential role of circuit-based biomarkers in guiding precision therapeutics.

Some studies have investigated the association of depressive symptoms, Aβ, and α-synuclein to a smaller extent. A systematic review and meta-analysis by Twait and colleagues (2024) [64], including nine CSF studies, eleven PET studies, and five plasma studies, did not find an association between Aβ42 and depression symptoms in over 10,000 older adults without dementia. Preliminary data show that α-synuclein has been associated with the severity of depression and might mediate depressive-like behavior [65,66]. However, studies investigating the association between these proteins and depressive and anxiety symptoms in PD are scarce. The present study evaluated over 1000 patients with PD who had these biomarkers measured in the CSF and found no correlation between depression and anxiety scores and these biomarkers. This is in line with previous work on healthy subjects, but is novel information regarding PD [64]. The results were consistent whether GDS lower (≥5) or higher (≥9) GDS, and STAI lower (≤40) or higher (>40) STAI cutoffs were used for depression and anxiety, respectively, regardless of using biomarkers as continuous or dichotomized variables.

α-synuclein and Aβ42 appear to be important markers of neuropathological staging, cognitive risk, and motor progression (as α-synuclein was significantly lower in the CSF of PD patients, not necessarily correlated with MDS-UPDRS part III scores, in line with previous literature [67]), but mood disturbances in PD appear to be driven chiefly by monoaminergic disruption, neuroinflammatory cascades, and psychosocial factors that operate on timescales and circuits only loosely related to bulk protein aggregation.

### 4.3. Strengths and Limitations

This work also has numerous strengths, including the large sample size. The fact that biomarkers were validated according to well-established associations with cognitive decline and PD, and that the results were very consistent, even after adjustment for confounders. Our study could detect and highlight important roles of CSF biomarkers in non-motor symptoms of PD and confirm other findings in the literature, mainly in cognitive decline. Aβ42-related biomarkers were significantly associated with lower MoCA scores. These results reinforce that, although useful biomarkers for other purposes, these molecules were not associated with depression or anxiety. To the best of our knowledge, this study integrated an approach of biomarkers, humor disorders, and cognition. The main limitation of the current study is that MoCA, GDS, and STAI are limited scales, mostly appropriate for screening, which do not encompass the entire complexity of these cognitive and mood disorders, even though they are widely used, standardized ways of quantifying symptoms.

## 5. Conclusions

In our cohort, cognitive performance (MoCA) was observed most closely onto tau-related pathology rather than α-synuclein or Aβ42 alone. Patients meeting with cognitive impairment demonstrated higher CSF p-tau and t-tau, as well as elevated p-tau/Aβ42 and t-tau/Aβ42 ratios. Although the individual tau measures lost statistical significance after adjustment for age and education, the tau/Aβ42 ratios remained robust, suggesting that the balance between tau phosphorylation and amyloid clearance. However, no association was observed between depression and anxiety symptoms and CSF biomarkers. Thus, while Aβ42 and α-synuclein remain useful for staging and cognitive risk, PD mood disturbances are probably driven by other monoaminergic dysregulation, neuroinflammation, and psychosocial factors. These results align with prior reports that tau species potentiate α-synuclein toxicity and accelerate synaptic dysfunction, while aggregated Aβ42 facilitates tau hyperphosphorylation, forming a pathogenic mechanism that impairs striatal and limbic circuits that are critical for executive and memory processes.

## Figures and Tables

**Table 1:** Baseline characteristics according to MoCA test.

	MoCA Test		
	≥26 (*n* = 304)	<26 (*n* = 134)	*p*-Value
Age	59.09 (±9.01)	64.28 (±8.96)	<0.001
Sex (Male)	179 (58.9%)	79 (59.0%)	1
White	301 (99.0%)	124 (92.5%)	<0.001
Hispanic/Latino	19 (6.2%)	8 (6.0%)	1
Asian	2 (0.7%)	4 (3.0%)	0.14
Black	0	3 (2.2%)	0.05
Education Years	16.21 (±3.34)	14.81 (±4.02)	<0.001
MDS UPDRS Part III	19.64 (±9.54)	19.58 (±9.83)	0.96
Total STAI	47.12 (±5.02)	46.66 (±6.32)	0.45
Total GDS	5.39 (±1.55)	5.85 (±1.68)	0.008
MoCA Test	28.01 (±1.37)	22.66 (±3.09)	<0.001
LRRK2	6 (5.6%)	8 (13.8%)	0.13

Data are presented as mean (±standard deviation) or frequency (valid %). *p*-values refer to *t*-tests (age and education) or chi-squared tests (sex and ethnicity). LRRK2: LRRK2 gene positive Parkinson’s Disease. MDS UPDRS: Movement Disorders Society Unified Parkinson’s Disease Rating Scale. MoCA: Montreal Cognitive Assessment. STAI: State-Trait Anxiety Inventory.

**Table 2:** Baseline characteristics according to depression screening.

	Depression Screening		
	Positive (GDS ≥ 5) (*n* = 349)	Negative (GDS < 5) (*n* = 96)	*p*-Value
Age	60.4 (±9.5)	62.0 (±8.2)	0.09
Sex (Male)	200 (57.3%)	63 (65.6%)	0.18
Ethnicity			
White	336 (96.3%)	95 (99.0%)	0.32
Hispanic/Latino	24 (6.9%)	4 (4.2%)	0.47
Asian	5 (1.4%)	1 (1.0%)	1
Black	4 (1.1%)	0	0.66
Other	8 (2.3%)	0	0.53
MDS UPDRS Part III	19.5 (±9.9)	20.1 (±8.9)	0.59
STAI	47.5 (±5.2)	45.1 (±5.9)	<0.001
MoCA Test	26.2 (±3.4)	26.9 (±2.2)	0.01
LRRK2	12 (9.0%)	2 (6.1%)	0.84
Education (Years)	15.6 (±3.7)	16.7 (±3.1)	0.003

Data are presented as mean (±standard deviation) or frequency (valid %). *p*-values refer to *t*-tests (age and education) or chi-squared tests (sex and ethnicity). GDS: Geriatric Depression Scale. LRRK2: LRRK2 gene positive Parkinson’s Disease. MDS UPDRS: Movement Disorders Society Unified Parkinson’s Disease Rating Scale. MoCA: Montreal Cognitive Assessment. STAI: State-Trait Anxiety Inventory.

**Table 3:** Baseline characteristics according to anxiety screening.

	Anxiety Screening		
	Positive (STAI > 40) (*n* = 395)	Negative (STAI ≤ 40) (*n* = 50)	*p*-Value
Age	60.6 (±9.3)	61.8 (±8.6)	0.35
Sex (Male)	231 (58.5%)	32 (64.0%)	0.55
Ethnicity			
White	382 (96.7%)	49 (98.0%)	0.95
Hispanic/Latino	25 (6.3%)	3 (6.0%)	1
Asian	5 (1.3%)	1 (2.0%)	1
Black	4 (1.0%)	0	1
Other	8 (2.1%)	0	0.93
Education Years	15.9 (±3.7)	15.2 (±2.7)	0.09
MDS UPDRS Part III	19.4 (±9.7)	21.9 (±9.3)	0.08
MoCA Test	26.5 (±3.1)	25.1 (±3.8)	0.02
LRRK2	12 (8.5%)	2 (8.3%)	1

Data are presented as mean (±standard deviation) or frequency (valid %). *p*-values refer to *t*-tests (age and education) or chi-squared tests (sex and ethnicity). LRRK2: LRRK2 gene positive Parkinson’s Disease. MDS UPDRS: Movement Disorders Society Unified Parkinson’s Disease Rating Scale. MoCA: Montreal Cognitive Assessment. STAI: State-Trait Anxiety Inventory.

**Table 4:** Biomarker levels for patients with Parkinson’s disease and controls.

	Healthy Subjects (*n* = 108)	Parkinson’s Disease (*n* = 300)	*p*-Value
Alpha-synuclein	119.7 (±51.6)	100.62 (±47.0)	<0.01
pTau	17.9 (±9.3)	14.1 (±5.0)	<0.001
tTau	195.0 (±83.7)	163.0 (±55.5)	<0.001
Aβ-42	1031.3 (±527.8)	923.0 (±398.3)	0.05
Aβ-42 < 1100	71 (65.7%)	217 (72.3%)	0.244
pTau/Aβ-42 > 0.022	14 (14.3%)	19 (6.9%)	0.05
tTau/Aβ-42 > 0.26	16 (15.2%)	21 (7.2%)	0.03

Data are presented as mean (±standard deviation) or frequency (valid %). *p*-values refer to *t*-tests.

**Table 5:** Biomarker levels according to MoCA scores.

	Overall (*n* = 300)	MoCA ≥ 26 (*n* = 213)	MoCA < 26 (*n* = 87)	*p*-Value
Alpha-synuclein	100.6 (±47.0)	98.7 (±42.3)	106.2 (±58.8)	0.46
Alpha-synuclein > p75	27 (17.2%)	20 (17.1%)	7 (17.5%)	1
pTau	14.09 (±4.97)	13.64 (±4.49)	15.1 (±5.8)	0.02
tTau	163.0 (±55.4)	158.1 (±51.9)	174.2 (±61.6)	0.01
Aβ-42	923.0 (±398.3)	928.6 (±388.4)	909.1 (±423.6)	0.71
Aβ-42 < 1100	217 (72.3%)	152 (71.4%)	65 (74.7%)	0.65
pTau/Aβ-42 > 0.022	19 (6.9%)	7 (3.6%)	12 (14.6%)	0.003
tTau/Aβ-42 > 0.26	21 (7.2%)	8 (3.9%)	13 (15.1%)	0.002

Data are reported as mean (±standard deviation). *p*-values refer to *t*-tests.

**Table 6:** Biomarker association with cognitive decline adjusted for age and education.

**Univariable**	**OR**	**95% Confidence Interval**		***p*-Value**
Alpha-synuclein	1.003	0.996	1.011	0.38
pTau	1.06	1.01	1.11	0.01
tTau	1.005	1.001	1.009	0.01
Aβ-42	0.999	0.999	1.000	0.70
Alpha-synuclein > p75	1.03	0.37	2.56	0.95
Aβ-42 < 1100	1.19	0.68	2.12	0.56
pTau/Aβ-42 > 0.022	4.53	1.75	12.6	<0.01
tTau/Aβ-42 > 0.26	4.40	1.78	11.5	<0.01
Multivariable *	**OR**	**95% Confidence Interval**		***p*-Value**
Alpha-synuclein	1.00	0.99	1.01	0.69
pTau	1.02	0.98	1.07	0.31
tTau	1.00	0.99	1.01	0.36
Aβ-42	0.999	0.998	1.00	0.35
Alpha-synuclein > p75	0.79	0.26	2.11	0.65
Aβ-42 < 1100	1.35	0.75	2.51	0.32
pTau/Aβ-42 > 0.022	4.64	1.67	13.8	<0.01
tTau/Aβ-42 > 0.26	4.18	1.60	11.5	<0.01

The regressions evaluated the association between biomarkers and cognitive decline (MoCA < 26). * Adjusted for age and education years.

**Table 7:** Biomarker levels according to depression scores.

	**Overall**	**GDS ≥ 5**	**GDS < 5**	***p*-Value**
Alpha-synuclein	100.62 (±47.0)	102.9 (±49.6)	94.5 (±39.2)	0.27
pTau	14.09 (±5.0)	14.0 (±4.8)	14.3 (±5.5)	0.62
tTau	163.0 (±55.5)	161.1 (±53.8)	169.5 (±60.5)	0.25
Aβ-42	924.2 (±397.8)	917.7 (±413.1)	946.1 (±343.7)	0.57
Alpha-synuclein > p75	27 (17.2%)	22 (19.3%)	5 (11.6%)	0.37
Aβ-42 < 1100	217 (72.3%)	167 (72.3%)	50 (72.5%)	0.99
pTau/Aβ-42 > 0.022	19 (6.9%)	16 (7.7%)	3 (4.5%)	0.53
tTau/Aβ-42 > 0.26	21 (7.2%)	17 (7.6%)	4 (5.9%)	0.83
	**Overall**	**GDS ≥ 9**	**GDS < 9**	***p*-Value**
Alpha-synuclein	100.62 (±47.0)	100.60 (±47.3)	101.39 (±33.4)	0.97
pTau	14.09 (±5.0)	14.14 (±5.0)	12.57 (±4.1)	0.20
tTau	163.0 (±55.5)	163.7 (±55.3)	146.85 (±58.3)	0.29
Aβ-42	924.2 (±397.8)	930.0 (±400.7)	770.4 (±286.0)	0.1
Alpha-synuclein > p75	27 (17.2%)	27 (17.5%)	0	0.98
Aβ-42 < 1100	217 (72.3%)	208 (72.0%)	9 (81.8%)	0.71
pTau/Aβ-42 > 0.022	19 (6.9%)	19 (7.2%)	0	0.87
tTau/Aβ-42 > 0.26	21 (7.2%)	21 (7.5%)	0	0.73

Data are reported as mean (±standard deviation). *p*-values refer to *t*-tests.

**Table 8:** Biomarker association with depression adjusted for age and education.

**Univariable**	**OR**	**95% Confidence Interval**		***p*-Value**
Alpha-synuclein	1.004	0.996	1.013	0.32
pTau	0.987	0.942	1.037	0.59
tTau	0.997	0.993	1.002	0.22
Aβ-42	0.999	0.999	1.001	0.60
Alpha-synuclein > p75	1.82	0.68	5.74	0.26
Aβ-42 < 1100	0.99	0.53	1.79	0.98
pTau/Aβ-42 > 0.022	1.79	0.57	7.86	0.37
tTau/Aβ-42 > 0.26	1.31	0.47	4.69	0.63
Multivariable *	**OR**	**95% Confidence Interval**		***p*-Value**
Alpha-synuclein	1.005	0.997	1.014	0.25
pTau	0.990	0.943	1.042	0.71
tTau	0.997	0.993	1.002	0.30
Aβ-42	0.999	0.999	1.001	0.60
Alpha-synuclein > p75	2.03	0.75	6.54	0.19
Aβ-42 < 1100	0.98	0.53	1.78	0.95
pTau/Aβ-42 > 0.022	2.03	0.64	9.08	0.28
tTau/Aβ-42 > 0.26	1.48	0.51	5.36	0.50

The regressions evaluated the association between biomarkers and positive depression screening (GDS ≥ 5). * Adjusted for age and education years.

**Table 9:** Biomarker levels according to anxiety scores.

	Anxiety Screening		
	Positive (STAI > 40) (*n* = 265)	Negative (STAI ≤ 40) (*n* = 35)	*p*-Value
Alpha-synuclein	100.7 (±47.7)	100.3 (±41.6)	0.97
Alpha-synuclein > p75	23 (16.4%)	4 (23.5%)	0.69
pTau	14.1 (±5.03)	14.2 (±4.6)	0.87
tTau	163.1 (±56.6)	162.6 (±47.3)	0.95
Aβ-42	922.0 (±404.8)	930.4 (±350.5)	0.90
Aβ-42 < 1100	192 (72.5%)	25 (71.4%)	1
pTau/Aβ-42 > 0.022	18 (7.5%)	1 (3.0%)	0.57
tTau/Aβ-42 > 0.26	20 (7.8%)	1 (2.9%)	0.48

Data are reported as mean (±standard deviation). *p*-values refer to *t*-tests.

## Data Availability

The original data presented in the study are openly available in Parkinson’s Progression Markers Initiative at http://ppmi-info.org/ (Accessed 2 August 2025).

## Supplementary Materials

The following supporting information can be downloaded at https://www.mdpi.com/article/10.3390/biomedicines13102478/s1, Figure S1. Cognitive and Affective Trajectories, Figure S2. Biomarker Trajectories
