# Supplementary material for: Dissociation of Clinical Outcomes and CSF Proteinopathy Biomarkers in Parkinson’s Disease: Cognitive–Affective Dissociation with Specificity for Tau
Source: Biomedicines. 2025 Oct 11;13(10):2478. doi: 10.3390/biomedicines13102478 (PMC12561310; doi:10.3390/biomedicines13102478)

Supplementary Material

Figure S1. Cognitive and Affective Trajectories …………………………………………… 2
Figure S2. Biomarker Trajectories ………………………………………………………… 3

Figure S1. Cognitive and Affective Trajectories

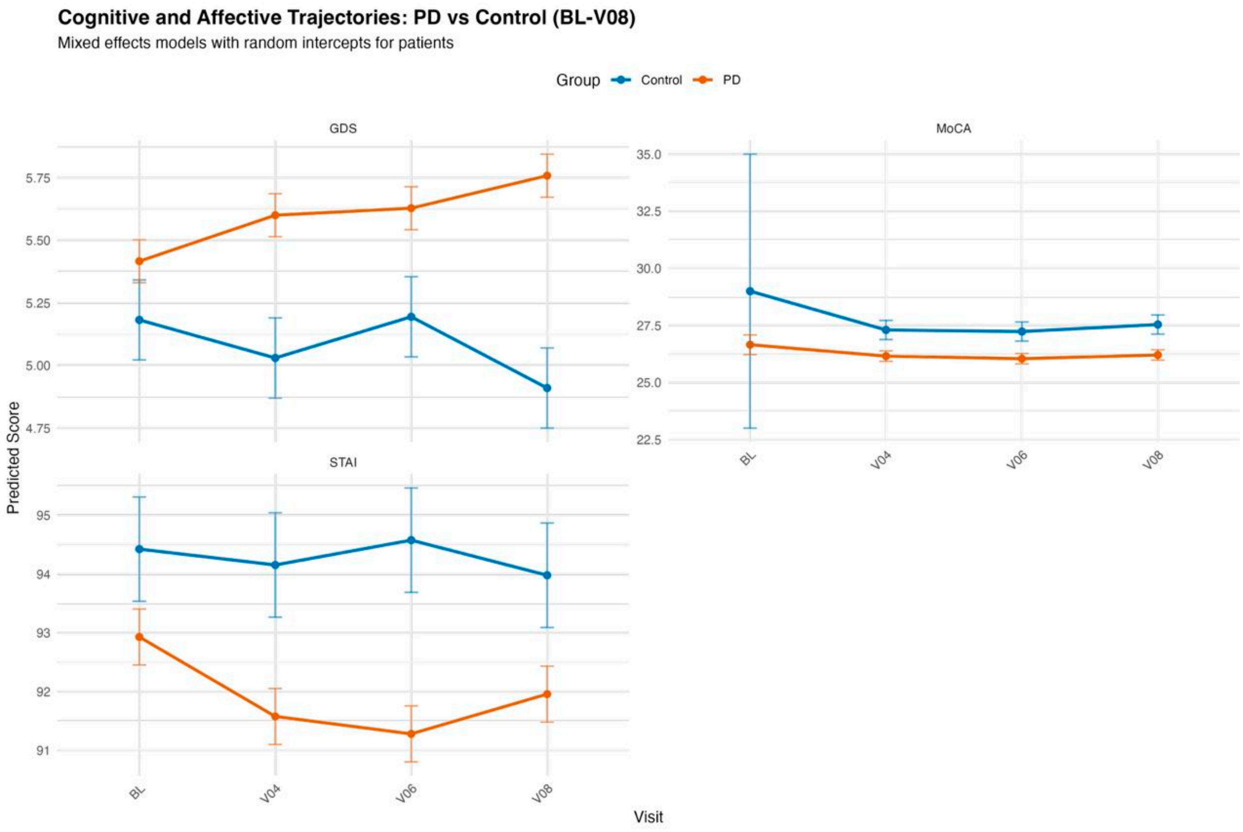

Figure S2. Biomarker Trajectories

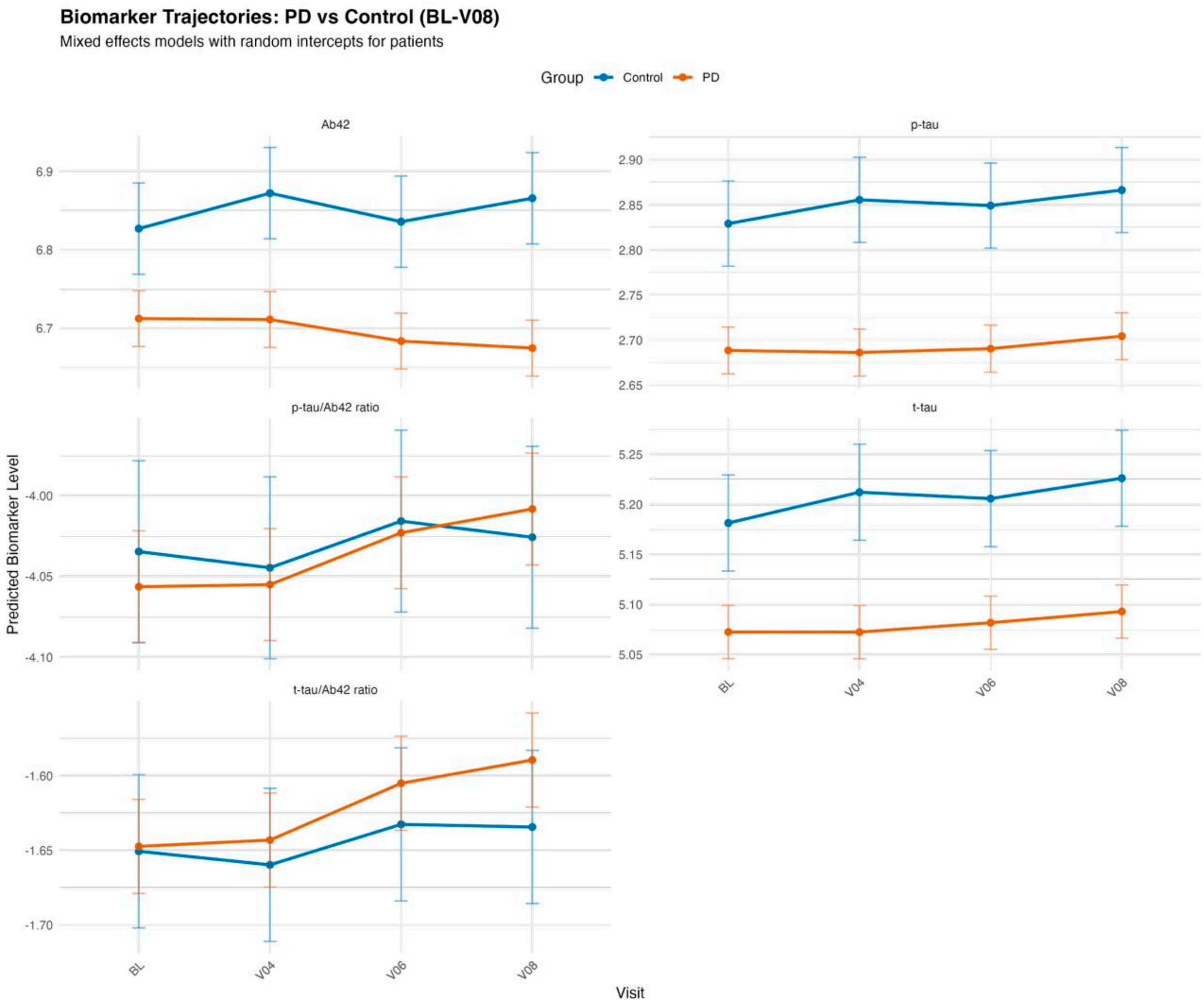

Supplement: Supplementary file 1 [file biomedicines-13-02478-s001.zip › biomedicines-3893857-supplementary.pdf]
